# An Extraordinary Case of Hemorrhage

**Published:** 1853-07

**Authors:** T. C. Harris

**Affiliations:** London.


### ARTICLE VII.

### *An Extraordinary Case of Hemorrhage.* By T. C. Harris, Esq., London.

A few years since I was sent for by two medical gentlemen, who had been trying to stop hemorrhage caused by the bungling extraction of a superior molar tooth; upon inquiry of them what course had been pursued, I was informed that several attempts had been made to divide the artery, but as the blood flowed so copiously this was entirely abandoned, as during the operation the lady was almost suffocated. Nitras argenti had also been applied two or three times, but previously the benzoin and mastic were used, both together and separately, all appeared to no purpose.

I immediately ordered some ice to be sent for, and to be kept continually applied in the mouth, to give me time to prepare an apparatus to effectually stop the bleeding. I took the model of the mouth in the usual way for making artificial teeth, struck up a large silver plate, sufficiently broad to cover the bruised part with a cavity, put on two hard gold clasps, one to the bicuspid the other to the second molar, with a pin in the centre to secure a piece of cork, which was cut to fit the inferior maxillary, as the patient had lost some of her under molar teeth, so as to cause some additional pressure to the plate when in the mouth. From the time I first saw the lady to the completion of this plate was nearly three hours, having cautioned them before I left not to expect me in less time. Now all was ready, I took with me my stopping instruments, and commenced filling the cavity with cotton dipped in the benzoin, then closed the clasps, put the plate in her mouth, recommending her at the same time to keep her jaws clenched for about half an hour, after which she might remove the cork which was only temporary; in less than five minutes the hemorrhage entirely ceased, much to the satisfaction of the lady's family and medical attendants.

I must say, that during the whole course of my practice, which is about twenty-five years, I have never met with a more formidable case. I saw the patient on the following day, removed the plate, and left her quite well.

